# Strategies for sample labelling and library preparation in DNA metabarcoding studies

**DOI:** 10.1111/1755-0998.13512

**Published:** 2021-10-13

**Authors:** Kristine Bohmann, Vasco Elbrecht, Christian Carøe, Iliana Bista, Florian Leese, Michael Bunce, Douglas W. Yu, Mathew Seymour, Alex J. Dumbrell, Simon Creer

**Affiliations:** ^1^ Faculty of Health and Medical Sciences Section for Evolutionary Genomics Globe Institute University of Copenhagen Copenhagen Denmark; ^2^ Department of Environmental Systems Science ETH Zurich Zürich Switzerland; ^3^ Department of Genetics University of Cambridge Cambridge UK; ^4^ Tree of Life Wellcome Sanger Institute Hinxton UK; ^5^ Aquatic Ecosystem Research Faculty of Biology University of Duisburg‐Essen Essen Germany; ^6^ Trace and Environmental DNA (TrEnD) Laboratory School of Molecular and Life Sciences Curtin University Perth WA Australia; ^7^ State Key Laboratory of Genetic Resources and Evolution Kunming Institute of Zoology Chinese Academy of Sciences Kunming China; ^8^ School of Biological Sciences Norwich Research Park University of East Anglia Norwich UK; ^9^ Center for Excellence in Animal Evolution and Genetics Chinese Academy of Sciences Kunming Yunnan China; ^10^ Department of Ecology Swedish University of Agricultural Sciences Uppsala Sweden; ^11^ School of Life Sciences University of Essex Colchester UK; ^12^ Molecular Ecology and Evolution Group School of Natural Sciences Bangor University Gwynedd UK

**Keywords:** amplicon sequencing, biodiversity assessment, eDNA, environmental DNA, high‐throughput sequencing, Illumina sequencing, library preparation

## Abstract

Metabarcoding of DNA extracted from environmental or bulk specimen samples is increasingly used to profile biota in basic and applied biodiversity research because of its targeted nature that allows sequencing of genetic markers from many samples in parallel. To achieve this, PCR amplification is carried out with primers designed to target a taxonomically informative marker within a taxonomic group, and sample‐specific nucleotide identifiers are added to the amplicons prior to sequencing. The latter enables assignment of the sequences back to the samples they originated from. Nucleotide identifiers can be added during the metabarcoding PCR and during “library preparation”, that is, when amplicons are prepared for sequencing. Different strategies to achieve this labelling exist. All have advantages, challenges and limitations, some of which can lead to misleading results, and in the worst case compromise the fidelity of the metabarcoding data. Given the range of questions addressed using metabarcoding, ensuring that data generation is robust and fit for the chosen purpose is critically important for practitioners seeking to employ metabarcoding for biodiversity assessments. Here, we present an overview of the three main workflows for sample‐specific labelling and library preparation in metabarcoding studies on Illumina sequencing platforms; one‐step PCR, two‐step PCR, and tagged PCR. Further, we distill the key considerations for researchers seeking to select an appropriate metabarcoding strategy for their specific study. Ultimately, by gaining insights into the consequences of different metabarcoding workflows, we hope to further consolidate the power of metabarcoding as a tool to assess biodiversity across a range of applications.

## 1 INTRODUCTION

In recent years, the analysis of environmental DNA (eDNA) and DNA extracted from bulk specimen samples has experienced an enormous surge in popularity in basic and applied biodiversity studies seeking to detect e.g., animal, plant, algae, fungi, and bacteria (Bálint et al., 2016; Compson et al., 2020; Creer et al., 2016; Jarman et al., 2018; Lindahl et al., 2013; Taberlet et al., 2012). Within the field of genetic biodiversity assessment, DNA metabarcoding is currently the most widely used approach, as it allows targeted, parallel, and as such relatively cost‐effective, identification of multiple taxa from environmental samples, such as soil, water, and faeces, as well as from bulk samples of organisms (Taberlet, Coissac, Pompanon, et al., 2012). Here, the applications of metabarcoding range widely; for example, detection of invasive species (e.g., Pochon et al., 2013); assessment of water quality via identification of freshwater invertebrates in bulk specimen samples (e.g., Elbrecht et al., 2017) and environmental samples (e.g., Seymour et al., 2020); identification of plant‐pollinator interactions (e.g, Gous et al., 2019; Lucas et al., 2018); detection of vertebrate wildlife via invertebrate “samplers” of vertebrate blood or faeces (e.g., Calvignac‐Spencer et al., 2013), and assessment of for example, niche partitioning (e.g., Razgour et al., 2011) and ecosystem services (e.g., Aizpurua et al., 2017) through detection of diet items. Furthermore, metabarcoding is explored for implementation in routine biomonitoring around the world (Aylagas et al., 2018; Li et al., 2018, 2019; Pont et al., 2018, 2021; Zizka et al., 2020; www.danubesurvey.org; www.syke.fi), and is an integral component of the proposals for the Next Generation of Biomonitoring programmes (Bohan et al., 2017).

**FIGURE 1:**
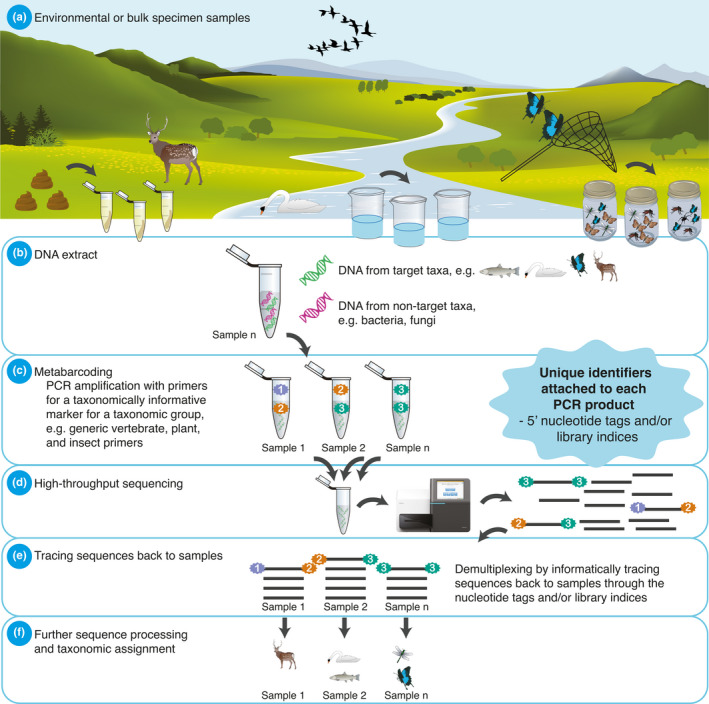
Simplified overview of a metabarcoding workflow. (a–b) DNA extracted from environmental samples such as soil, water, and faeces or from bulk specimen samples. The DNA extracts are typically a complex mix of DNA from target and nontarget organisms. (c) DNA extracts are PCR‐amplified with metabarcoding primers that target a taxonomically informative marker for a taxonomic group. Importantly, identifiers unique to each PCR product are added in the form of 5ʹ nucleotide tags on primers and/or as indices added to sequence libraries during library build. (d) The taxonomic markers of hundreds to thousands of samples are sequenced in parallel on a high‐throughput sequencing platform producing millions of sequence reads. (e) The sequences can be traced back to the samples they originated from through the nucleotide tags and/or library indices, and (f) can be further analysed. Images courtesy of the Integration and Application Network, University of Maryland Centre for Environmental Science (ian.umces.edu/symbols/) and Illumina.com

**FIGURE 2:**
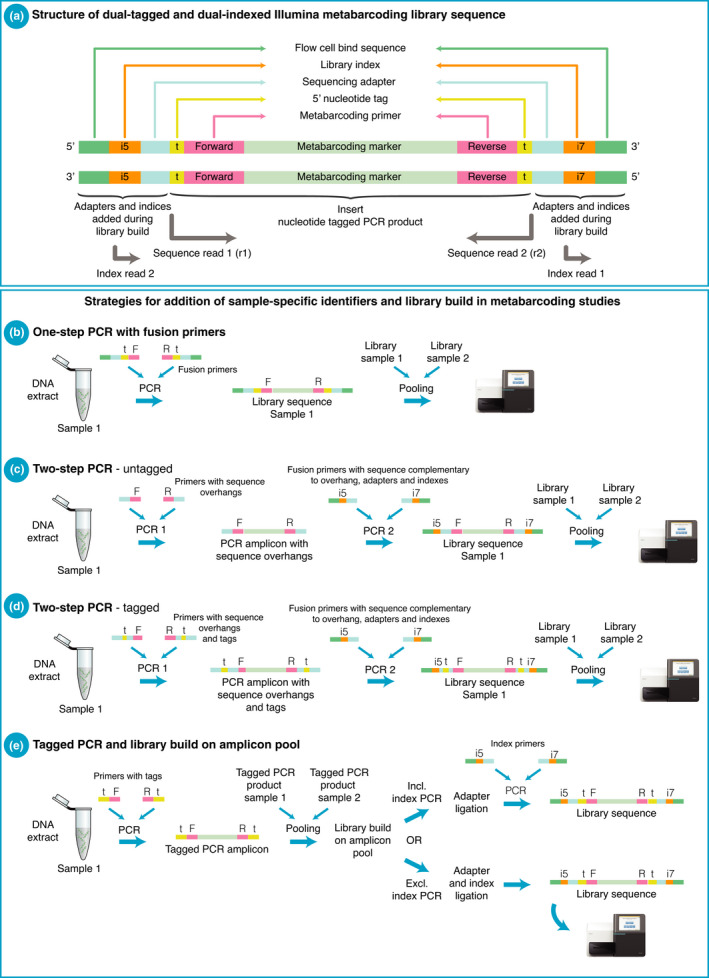
Metabarcoding approaches can be divided into three overall strategies for adding nucleotide tags and library indices. (a) The composition of a dual‐tagged and dual‐indexed metabarcoding Illumina library sequence. Note that the metabarcoding marker, primers, and tags are sequenced as Illumina read 1 and read 2, while index reads are sequenced separately as i7 and i5 reads and used to multiplex sequencing libraries. (b–e) Strategies for adding nucleotide tags and indices to metabarcoding markers. The one‐step PCR (b) is depicted with the use of nucleotide tags, which eliminates the need for indices

Metabarcoding relies on PCR amplification of extracted DNA with primers designed to target a taxonomically informative marker for a selected taxonomic group (Taberlet, Coissac, Pompanon, et al., 2012) (Figure 1). The backbone of metabarcoding analyses is the addition of sample‐specific nucleotide identifiers to amplicons and the use of these to assign metabarcoding sequences back to the samples they originated from (“demultiplexing”). This allows pooling of hundreds to thousands of samples for sequencing and utilisation of the capacity of high‐throughput sequencing platforms (Figure 1). Amplicon labelling can be achieved at two stages during a metabarcoding workflow: prior to library build, as 5′ nucleotide “tags” on metabarcoding primers, and during library build as library indices. The strategies to achieve this labelling can be categorised into three main approaches; one‐step PCR, two‐step PCR, and tagged PCR (Figure 2). All three approaches have advantages, challenges, and limitations which, if not considered, can result in misleading data interpretation, and in the very worst case can lead to unusable data and considerable wasted time and money, as for instance in the case of the so‐called “tag‐jumps” (Carøe & Bohmann, 2020; Esling et al., 2015; Schnell et al., 2015). Despite this, in contrast to discussions on metabarcoding substrate selection, DNA extraction, and data processing, the strategies for amplicon labelling and library preparation workflows have received little systematic attention in the metabarcoding literature (although see Murray et al., 2015).

Here, we present an overview of the three most commonly used workflows with which to achieve sample‐specific labelling and library preparation in metabarcoding studies, and how they can potentially influence the resulting data. For the sake of simplicity, we mainly focus on metabarcoding of plants and animals in basic and applied biodiversity studies with sequencing on arguably the most used high‐throughput sequencing platform series today, the Illumina sequencing platforms. Note that points raised will be relevant for metabarcoding of other organisms and to high‐throughput sequencing platforms with similar labelling structures to Illumina platforms, such as Ion Torrent (Thermo Fischer Scientific), BGI platforms (BGI Genomics), Oxford Nanopore Technologies MinION, and PacBio (Pacific Biosciences). In the present article, we provide critical considerations for researchers to choose the optimal metabarcoding strategy for generating reliable data tailored to their individual study; for example, regarding sample type and number, research question, speed of laboratory processing, contamination risk, budget, and whether similar studies are to be carried out in the laboratory. Ultimately, by gaining detailed and critical insights into the consequences of choosing different metabarcoding workflows, we hope to further increase the potential of metabarcoding as a reliable tool for use across a wide range of applications.

## 2 TAGGING AND INDEXING APPROACHES IN METABARCODING STUDIES

Today, the most commonly used high‐throughput sequencing platform for metabarcoding studies is the Illumina series, where for example the MiSeq, iSeq, HiSeq, NextSeq, and NovaSeq have been employed (Jarman et al., 2018). These platforms offer high throughput, relatively low error rates, and relatively long paired‐end reads, typically up to 150 bp of each paired read on the iSeq100, NextSeq550/1000/2000, HiSeq 3000/4000, and NovaSeq (up to 250 bp on SP flow cell), and 300 bp of each paired read on the MiSeq platform (www.illumina.com, applied in e.g., Elbrecht et al., 2017; Hope et al., 2014; Quéméré et al., 2013; Shehzad, Riaz, et al., 2012; Singer et al., 2019; Stoeck et al., 2018).

The sequencing depth required per sample is commonly much lower in metabarcoding studies than in shotgun sequencing studies (e.g., Srivathsan et al., 2015; Stat et al., 2017), and in metabarcoding studies it is (economically) feasible to sequence tens, hundreds, or even thousands of samples per sequencing run. To allow pooling and parallel sequencing of this magnitude, different molecular labelling systems have been developed. For metabarcoding studies, the addition of sample‐specific identifiers to PCR amplicons can be achieved either as nucleotide tags during the metabarcoding PCR, or as library indices when converting amplicons into sequencing libraries, that is, as part of the workflow of adding sequencing adapters to amplicons. A metabarcoding sequencing library consists of amplicons carrying sequencing adapters and indices and can consist of one or more PCR products from one or more samples as outlined below. Note that given the inconsistent use of terminology in the metabarcoding literature, for clarity, we use the original term for nucleotide tags in amplicon sequencing as used by Binladen et al., (2007), and Illumina's terminology to describe the nucleotide reads that are used to demultiplex sequencing libraries, the i5 and i7 index reads. That is, 5′ nucleotide tags are sequenced with the metabarcoding marker and primers in the Illumina sequencing read 1 (and read 2 for paired‐end sequencing), while library indices are sequenced as separate index reads, i.e., if dual‐indexing is performed as i5 and i7 reads (Figure 2a) (https://support.illumina.com).

Metabarcoding approaches can be divided into three overall strategies for adding nucleotide tags and library indices (Taberlet et al., 2018) (Figure 2):

1. The “one‐step PCR” approach in which sample DNA extracts are amplified and built into sequence libraries in one reaction. Here, metabarcoding primers carry sequencing adapters, nucleotide tags, and/or library indices, referred to as “fusion primers” (Figure 2b). This approach is used in for example, Kozich et al. (2013), Elbrecht and Leese (2015), Sickel et al. (2015), Grealy et al. (2016), Berry et al. (2017), Elbrecht et al. (2017), Hardy et al. (2017), Elbrecht and Steinke (2018), Seersholm et al. (2018), and Bessey et al. (2020). If indices are used, then each PCR replicate or sample is a sequencing library and as such is returned as a separate fastq file following sequencing. It should be noted that most studies add nucleotide tags next to the primers thereby eliminating the need for i5 and i7 “indexing”.
2. The two‐step PCR approach in which sample DNA extracts are PCR‐amplified with two primer sets. In the primary reaction the metabarcoding primers carry 5′ sequence overhangs of c. 33–34 nucleotides in length. These can be with (Clarke et al., 2017; Griffiths et al., 2020; Kitson et al., 2018; Li et al., 2019; Vesterinen et al., 2018) or without (Bista et al., 2017; de Vere et al., 2017; Galan et al., 2017; Miya et al., 2015; Swift et al., 2018; Vesterinen et al., 2018) nucleotide tags (Figure 2c,d). The sequence overhangs allow the resulting amplicons to be targeted by the second round of primers, which carry sequencing adapters and indices. Most commonly, two consecutive PCRs are carried out, such as in Miya et al. (2015), de Vere et al. (2017), Galan et al. (2017), Kaunisto et al. (2017), Swift et al. (2018), and Vesterinen et al. (2018). However, a few studies carry out only one reaction with the two primer sets, such as Clarke, Czechowski, et al. (2014). The two‐step PCR approach is based on Illumina's 16S rRNA system originally developed for microbiome studies (www.illumina.com). If unique ndexing is used on PCR replicates in the two‐step approach, each PCR replicate is an individual sequencing library and as such is returned as a separate fastq file following sequencing.
3. The “tagged PCR” approach, in which sample DNA extracts are PCR amplified with metabarcoding primers that carry 5′ nucleotide tags. Following PCR amplification, the individually tagged PCR products are pooled, and ligation‐based library preparation is carried out on pools of 5′ tagged amplicons. The ligated adapters can themselves contain indices, which eliminates the need for a second PCR step (e.g., Carøe & Bohmann, 2020; Thomsen et al., 2016), or the adapter ligation can be followed by a PCR step with indexed primers (e.g., Bohmann et al., 2018; Hope et al., 2014). This approach was first demonstrated by Binladen et al. (2007) on the 454 FLX platform and has since been used in for example, Shehzad, McCarthy, et al. (2012), Hibert et al. (2013), Hope et al. (2014), Thomsen et al. (2016), Apothéloz‐Perret‐Gentil et al. (2017), Sigsgaard et al. (2017), Bakker et al. (2017), Kocher et al. (2017), Thomsen and Sigsgaard (2019), and Lynggaard et al. (2020) (Figure 2e). In this approach, each library pool of PCR replicates is a sequencing library and is returned as a separate fastq file, each of which can contain data from a large number of tagged PCR replicates.

For all three strategies, it is important to carefully design tags and indices to ensure that oligonucleotide synthesis, PCR, and sequencing error will not cause them to be unidentifiable or confused (Coissac, 2012; Faircloth & Glenn, 2012). Further, all three strategies offer the option to add extra nucleotides to shift PCR amplicons in relation to each other and thereby to increase sequence complexity on the flow cell (“heterogeneity spacers”, see for example, Bohmann et al., 2018; De Barba et al., 2014; Elbrecht & Leese, 2015).

In this article, we discuss the three main metabarcoding strategies. One approach not mentioned here is library preparation on individual unlabelled PCR products through a ligation‐based library preparation protocol with or without an index PCR step. However, such ligation based protocol would entail several protocol steps to be carried out on each PCR product, such as end‐repair and ligation of adapters (e.g., carrying indices such as in Illumina's TruSeq Nano DNA Library Prep kit, see Zizka et al., 2019). The reason that we do not consider this approach a main metabarcoding strategy is due to low reported use of this method, its high cost and workload and thereby limited throughput (Zizka et al., 2019).

## 3 PROS AND CONS OF METABARCODING APPROACHES

The ability to tag and index amplicons to fully harvest the power of high‐throughput sequencing comes at a price as the labelling and pooling of hundreds of PCR replicates is highly complex and entails costs associated with preventing, detecting, and eliminating errors and biases. None of the metabarcoding approaches presented here is perfect; rather each of them has pros and cons. Below, we outline the advantages and disadvantages, specifically addressing issues related to cross‐contamination risk, PCR amplification efficiency, chimera formation, tag‐jumping, index‐misassignment, cost, and workload. The issues associated with each metabarcoding strategy are important to keep in mind for choosing a metabarcoding strategy and for designing laboratory workflows and interpreting results.

### 3.1 Cross‐contamination risk

During the metabarcoding PCR, here specified as the PCR in which the metabarcoding marker is targeted, relatively short DNA sequences (typically <350 bp) are enriched through amplification. Especially when targeting trace amounts of DNA, PCR amplification can be highly susceptible to contamination and thereby to false positives. The risk of contamination when preparing metabarcoding PCRs, that is from the surroundings or laboratory reagents, is the same no matter which of the three overall metabarcoding approaches is used. Moreover, regardless of the metabarcoding strategy employed, cross‐contamination can happen between nucleotide tagged and indexed primer stocks (which are delivered at high molarity). The risk of this happening will be similar between the strategies and will depend on the number of samples and the chosen setup within the employed strategy. In the following, we will therefore focus on how the three main metabarcoding approaches differ in their ability to allow detection of cross‐contamination between PCR products after the metabarcoding PCR.

PCR products are labelled during the metabarcoding PCR amplification in the one‐step PCR approach (Figure 2b), the two‐step PCR approach where tagging is carried out in the first PCR (Figure 2d), and the tagged PCR approach (Figure 2e). If the resulting PCR products carry different tag combinations then cross‐contamination between them is obviously not of concern. However, if the same tag combinations occur across multiple samples, then cross‐contamination between them can be an issue. A solution is to process them in separate batches to avoid cross‐contamination. Some laboratories do not reuse tag‐primer combinations to eliminate cross‐contamination risk (see Murray et al., 2015).

In the two‐step approach, sample‐specific labelling is not necessarily carried out during the metabarcoding PCR (Figure 2c,d). If not labelled, there is a risk of cross‐contamination between unlabelled PCR products when handling them prior to the second PCR (Zizka et al., 2019). Therefore, this metabarcoding approach has the greatest theoretical risk of cross‐contamination between PCR products (Figure 2c, Table 1). The risk of this kind of cross‐contamination is eliminated if tagging is carried out in the first PCR, see for example Kitson et al. (2018). If untagged metabarcoding primers are used in the two‐step PCR approach (Figure 2c), then cross‐contamination can be eliminated if the two PCRs are carried out in the same reaction, that is, both two primer sets are included, see for example Clarke, Czechowski, et al. (2014).

**TABLE 1:** Features of the three main metabarcoding strategies

Feature	Metabarcoding strategy				
	One‐step PCR	Two‐step PCR		Tagged PCR	
	With 5′ nucleotide tags, without i5 and i7 indices	Without 5′ nucleotide tags on metabarcoding primers	With 5′ nucleotide tags on metabarcoding primers	Library preparation with T4 DNA polymerase blunt‐ending and post‐ligation PCR	Library preparation without T4 DNA polymerase blunt‐ending and post‐ligation PCR
Handling and workload	**↓**	**↑**	**↑**	**↑**	**↑**
Risk of cross‐contamination between PCR products	**↓**	**↑**	**↓**	**↓**	**↓**
Tag‐jumps	No	No	No	Yes	No
Potential for index misassignment/library bleeding on the flow cell	No (only if indices are used)	Yes	Yes (if indices are used)	Yes	Yes
Decrease in PCR efficiency due to nucleotide additions to metabarcoding primers	High	Potentially high	Potentially high	Low	Low
Cost of metabarcoding primers	**↑**	**↓**	**↑**	**↓**	**↓**
Number PCR steps prior to sequencing	1	2	2	2	1

Irrespective of the chosen approach, cross‐contamination can be detected and filtered out by including sample replicates, PCR replicates, and positive and negative controls. Thus, these should be included in the laboratory workflow and sequencing (e.g., Bista et al., 2017). An important measure that enables one to filter out potential contamination during data processing is to use different nucleotide tag or library index combinations on each sample's individual PCR replicates. This will allow for stringent sequence processing across each sample's PCR replicates, that is, a restrictive approach in which only sequences that are shared by a number of a sample's PCR replicates are retained (see Alberdi et al., 2018, applied in, for example, Giguet‐Covex et al., 2018, 2014; De Barba et al., 2014; Hope et al., 2014; Cohen et al., 2020; Lynggaard et al., 2021; Yang et al., 2021).

### 3.2 PCR amplification

PCR amplification introduces biases, such as primer biases, and errors, such as nucleotide substitutions and chimeras (Haas et al., 2011; Murray et al., 2015; Piñol et al., 2015; Polz & Cavanaugh, 1998). Two of the three main metabarcoding strategies allow practitioners to carry out only a single PCR step before sequencing, namely the one‐step PCR approach and the tagged PCR approach in which PCR‐free library building is carried out (Figure 2b,e, Table 1). Because an extra PCR step adds an additional risk of introducing errors, these two approaches offer an advantage over the two‐step PCR method (Figure 2c,d) and the tagged PCR approach in which the workflow includes an index PCR step (Figure 2e). It should be noted that the number of cycles in the indexing PCR is typically kept low to minimize PCR errors (e.g., eight cycles: Bohmann et al., 2018). Throughout any of these workflows there is a need to keep PCR cycles to a minimum, which might be especially true of metabarcoding workflows with two PCR steps.

Aside from minimizing the number of PCR steps, the effect of 5′ nucleotide additions to metabarcoding primers should be considered as they are likely to decrease PCR efficiency (Murray et al., 2015; Schnell et al., 2015). Bulk sample and eDNA extracts consist of complex mixtures of DNA from a large number of organisms, which especially in the case of eDNA can be degraded (Taberlet et al., 2012). With DNA extracts, the primers are faced with the task of amplifying (trace copy number) target DNA from different taxa (Taberlet, Coissac, Pompanon, et al., 2012) potentially distorted by primer biases, inhibitors, and potentially abundant predator or host DNA (e.g., Clarke, Soubrier, et al., 2014; Deagle et al., 2014; Murray et al., 2015). Therefore, it is important to take the effect of 5′ nucleotide additions to metabarcoding primers into account.

The three main metabarcoding strategies have different lengths of nucleotide additions on the 5′‐end of metabarcoding primers. The longest 5ʹ‐nucleotide additions are found in the one‐step PCR approach where up to 60 nucleotides (sequence adapters and tags) are added to one or both of the primers, making the complete primer often over 80 bp long (e.g., Elbrecht & Leese, 2015). In the two‐step PCR approach (Figure 2c,d), the sequence overhangs on the metabarcoding primers used in the first PCR are approximately half the length of the fusion primers, for example, 33–34 nucleotides if using Illumina Nextera Indices. The tagged PCR approach has the shortest nucleotide additions to the metabarcoding primers (Figure 2e) with tags of typically 5–10 nucleotides in length (e.g. Alberdi et al., 2018; Coissac, 2012; De Barba et al., 2014). The long additions to the metabarcoding primers in the one‐step PCR approach cause a decrease in PCR efficiency, as witnessed by an increase in C_T_ values (Murray et al., 2015). A comparison of PCR efficiency to other metabarcoding strategies has not, to our knowledge, been formally assessed for the two‐step PCR approach, but the two‐step PCR approach has been shown to have higher consistency as compared to the one‐step fusion primer approach (Zizka et al., 2019). Even the short nucleotide additions in the tagged PCR approach have been shown to decrease PCR efficiency, as witnessed by a significant increase in C_T_ values (Schnell et al., 2015). Thus, no method is free of decreased PCR efficiency caused by the nucleotide additions to 5′‐end of metabarcoding primers. However, it has to our knowledge, not been formally tested whether ‐ and to what extent ‐ the shorter nucleotide tag additions in the tagged PCR approach offers greater PCR efficiency and taxonomic detection than the two other approaches, and thereby it can only be speculated that it is the most sensitive when it comes to detection of taxa in low abundance. It should be noted that increasing the cycle number in the PCR amplifications is not an acceptable solution to increase sensitivity, as increased cycle number will reduce taxonomic diversity (Kelly et al., 2019; Piñol et al., 2015). Regardless of metabarcoding strategy, we stress the importance of optimising PCR amplifications (usually by qPCR) to detect PCR inhibition, identify samples with low template quantity, and track PCR efficiency issues (Murray et al., 2015; Yang et al., 2021).

Theoretically, the reduced PCR efficiency in the one‐step and two‐step PCR approaches caused by the long overhangs on primers might be counteracted by spiking the PCRs with metabarcoding primers without any 5ʹ attachments (e.g., Murray et al., 2015). However, this has been shown to have modest PCR efficiency improvements for the one‐step approach (e.g., Murray et al., 2015). Alternatively, a pre‐enrichment can be carried out before the metabarcoding PCR. That is, running a PCR with metabarcoding primers with no nucleotide additions prior to the metabarcoding PCR, as done in Zizka et al. (2019) and Elbrecht and Steinke (2018) for the one‐step PCR approach. However, this not only introduces another PCR amplification step, but can increase the risk of cross‐contamination between PCR products due to the initial unlabelled PCR amplification step (e.g., Murray et al., 2015). Note that adding such a pre‐enrichment step to the one‐step approach can cause it to be mistaken for a two‐step PCR approach.

Apart from the length of the nucleotide additions, it has been investigated whether differences in nucleotide tag sequences can result in biases in the tagged PCR approach. Although some studies show that such tag bias is an issue (Berry et al., 2011; O’Donnell et al., 2016), other studies show that it is not (Leray & Knowlton, 2017; Yang et al., 2021). If tag bias does exist, it should theoretically be minimised if different tags are used on each sample's PCR replicates.

### 3.3 Chimeras and tag‐jumps

Chimeras can be formed during all PCR steps in any metabarcoding workflow (Figure 2b–e). Chimeras are amplicons which combine sequences from two or more different template molecules, and the majority are thought to result from incomplete primer extension during the elongation phase of the PCR cycle (Judo et al., 1998; Meyerhans et al., 1990; Shin et al., 2014; Wang & Wang, 1997). The probability of chimera formation increases when similar template sequences are amplified in the same PCR reaction ( Judo et al., 1998, Smyth et al., 2010, but see also Fonseca et al., 2012), such as during the metabarcoding PCR or during the index PCR amplification of pools of tagged amplicons (Figure 2e). There are different consequences of chimeric sequences depending on where they arise. If they are created during a PCR amplification of a single sample's DNA extract, the chimeras will be intrasample chimeras, which can be falsely interpreted as novel taxa and erroneously inflate measures of diversity. On the other hand, if chimeras are created during a PCR amplification of pooled tagged amplicons, such as in the tagged PCR approach (Figure 2e), the chimeras may be intersample chimeras. Such intersample chimeras can result in tag‐jumps and false attribution of amplicon sequences to samples, which can lead to false positives and inflation of diversity (Schnell et al., 2015).

All metabarcoding approaches are prone to intra‐sample chimeras. However, as chimera formation increases when similar sequences are amplified in the same PCR reaction (e.g. Judo et al., 1998; Smyth et al., 2010), the use of metabarcoding primers with long 5′ overhangs, as in the one‐step and two‐step approaches, might be more prone to chimera formation since they carry long and similar sequences at the 5ʹ end of the primers. However, this hypothesis requires testing. Intrasample chimeras can be reduced by limiting the number of PCR cycles and extending elongation time (Haas et al., 2011; Qiu et al., 2001). Also, if samples are subjected to multiple, independent PCRs, chimeras can be filtered out by keeping only sequences that occur in multiple PCR replicates, the “restrictive approach” described in Alberdi et al., (2018). Chimera detection programmes such as UCHIME (Edgar et al., 2011) can be used for further clean‐up.

Inter‐sample chimeras can cause havoc in metabarcoding studies. They can only occur in the tagged PCR approach where library build is carried out on pooled tagged amplicons from different samples (Figure 2e, Table 1). Here, tag‐jumps can create sequences with new combinations of the nucleotide tags used in the amplicon pool (Schnell et al., 2015). If the new combinations of tags are already used in the amplicon pool, it will cause false assignment of sequences to samples, which should be avoided at all cost (Esling et al., 2015; Schnell et al., 2015). Such tag‐jumps can cause negative controls to accumulate a number of sequences following bioinformatic sorting of sequences to samples, which makes sequencing of negative controls a valuable tool to detect tag‐jumps.

The rate of tag‐jumping has been estimated from ca. 2% to up to 49% of total sequences (Carøe & Bohmann, 2020; Esling et al., 2015; Schnell et al., 2015). This broad range can be caused by factors affecting intersample chimera formation during the index PCR. For example, DNA template and primer concentration, PCR cycle number, and sequence similarity (e.g., Carøe & Bohmann, 2020; Judo et al., 1998; Smyth et al., 2010). The range of tag‐jump proportions highlights the unreliability of including an index PCR step in the tagged PCR approach. It should be noted that tag‐jumps can also occur due to T4 DNA polymerase activity in the blunt‐ending step during library preparation, as demonstrated in library building for the Roche/454 sequencing platform (van Orsouw et al., 2007; Palkopoulou et al., 2016) and for the Illumina sequencing platform (Carøe & Bohmann, 2020).

To avoid tag‐jumps in the tagged PCR approach, and thereby prevent false assignment of sequences to samples, it is important to refine index PCR parameters to decrease the likelihood of chimera formation, or better yet, to omit the index PCR step (Figure 2e). Furthermore, blunt‐ending using T4 DNA polymerase should be circumvented during library preparation (Carøe & Bohmann, 2020; Palkopoulou et al., 2016; Schnell et al., 2015). If both T4 DNA polymerase blunt‐ending and index PCR are eliminated during library preparation of pools of tagged amplicons, tag‐jumps can practically be eliminated (Carøe & Bohmann, 2020).

If the library preparation protocol contains a T4 DNA blunt‐ending step and/or an index PCR step, and thereby can be assumed to generate tag‐jumps, they can be detected and removed by using “twin‐tags” during the original PCRs (e.g., F1‐R1, F2‐R2, etc.), because tag‐jumped sequences would then produce nontwinned tag combinations not used in the set‐up (e.g., F1‐R2, F2‐R3, etc.) (e.g. Schnell et al., 2015; Yang et al., 2021). However, using twin tags comes at the price of buying many more versions of tagged primers and building more libraries (Schnell et al., 2015). If twin tags are not used, chimera removal software can remove some chimeric sequences carrying false combinations of used tags (Schnell et al., 2015).

The extent of tag‐jumping and spillover of taxa between samples can be detected through inclusion of positive controls consisting of synthetic oligos or taxa not expected to occur in the data set. However, note that such controls do not enable confident elimination of false positives caused by tag‐jumps. The extent of tag‐jumping can also be assessed by comparing all observed combinations of used tags to all originally used tag combinations (Schnell et al., 2015; Zepeda Mendoza et al., 2016).

### 3.4 Misassignment of library indices

Incorrect assignment of indices between pooled libraries can cause sequence reads to be incorrectly assigned to libraries. Misassigned indices have been attributed to the formation of mixed clusters on the sequencing flow cell, that is, clusters originating from two different template molecules or clusters growing into each other, to low levels of free index primers present in the sequence library and to bulk amplification of pooled libraries (Costello et al., 2018; Nelson et al., 2014; Sinha et al., 2017; Valk et al., 2019; Vodak et al., 2018). Regardless of how index misassignment occurs, if it occurs in metabarcoding studies it can cause incorrect assignment of amplicon sequences to libraries, which can cause incorrect assignment of sequences to samples and false positives. This phenomenon can affect metabarcoding approaches that include indexing of libraries (Figure 2, Table 1). To avoid index misassignment it is recommended to dual‐index libraries with unique library index combinations (Kircher et al., 2012; Sinha et al., 2017), www.illumina.com). Further, stringent bead purification (or size selection) can remove free adapters/primers from the libraries (Owens et al., 2018). The labelling in the different metabarcoding approaches further allows for accounting for potential incorrect assignment of sequences to libraries. In the tagged PCR approach, unique tagging of PCR replicates across all pooled libraries can be used to account for (and detect) index misassignment. However, this can be costly. In the one‐step PCR approach, it is common to eliminate the use of i7 and i5 library indices, instead relying on 5′ nucleotide tags, which creates a single library that is free of index misassignment (Table 1). As with tag‐jumping, the extent of incorrect assignment of indices and spillover of taxa between samples can be detected through inclusion of positive controls consisting of taxa not expected to occur in the data set and by comparing all observed to all used combinations of used indices when demultiplexing libraries.

It is important not to mistake tag‐jumping, index misassignment, or cross‐contamination between PCR products with cross‐contamination of the primers themselves. Due to the high concentration of primers upon synthesis, cross‐contamination (e.g., by aerosols) can manifest itself as low numbers of sequence reads and could be misinterpreted as tag‐jumps or index‐bleeding. Due to the risk of primer cross‐contamination, some laboratories avoid ordering primers in 96‐well plates. As mentioned, the risk of cross‐contamination between nucleotide tagged primer stocks and indexed primer stocks, which could for example occur during resuspension of primers, will generally be the same no matter which of the three overall metabarcoding approaches is used. If the first PCR step in the two‐step PCR approach is carried out without tags (Figure 2c), the primers are unlabelled and any cross‐contamination between the primers will not have consequences.

### 3.5 Cost

Metabarcoding primers in the tagged and one‐step PCR approaches are labelled, whereas the metabarcoding primers in the two‐step approach can be either labelled or not (Figure 2). Due to the different labelling systems in the three primary metabarcoding approaches, there are different costs associated with them.

The fusion primers for the one‐step PCR approach are the most expensive metabarcoding primers amongst the three approaches. This is because differently labelled versions are purchased for each metabarcoding primer set and because the increased oligo length results in lower yield of the full length product. If indexing is used instead of tagging and unique matching indices are used to account for index misassignment, one‐step PCR can become increasingly expensive for larger scale studies. However all of this needs to be factored against the potential cost of repeating runs due to artefacts and contamination, and the fact that only a single PCR step is needed to go from sample extract to library. The tagged two‐step PCR primers will be the second‐most expensive (Figure 2d) due to their length and individual labelling.

In the tagged PCR approach (Figure 2e), the metabarcoding primers are relatively inexpensive as they only add 5′ tags of 5–10 nucleotides in length. However, these need to be purchased in many tagged versions for each metabarcoding primer set. Furthermore, if tag‐jumping is to be taken into account by only using each tag once in a library amplicon pool, for example, by only amplifying with twin forward and reverse tags, then metabarcoding primer sets have to be ordered in many differently labelled versions (Schnell et al., 2015). To keep costs down, this twin‐tagging needs to be balanced by pooling fewer PCR products into each library and thereby creating more sequence libraries, but this then increases expenses to library preparation (Figure 2e). However, if a library preparation protocol is used that does not create tag‐jumps, tags can be freely combined, which lowers the number of tagged primers that must be purchased (Carøe & Bohmann, 2020; Schnell et al., 2015). In contrast to the other two metabarcoding approaches, the tagged PCR approach includes ligation‐based library preparation of pools of amplicons, and the cost of this therefore has to be taken into account. The cost can be kept low if a protocol that does not generate tag‐jumps is used and only a few libraries have to be made.

If a large number of metabarcoding primer sets are used, the two‐step approach, where primers in the first PCR do not carry tags (Figure 2c), offers a relatively inexpensive solution. This means that the same primer set can be used across multiple samples and projects. This has the benefit that trying out new metabarcoding primer sets does not entail buying many labelled versions of the metabarcoding primer sets, as it does in the other metabarcoding approaches (Figure 2b,d,e). However, the second primer set in the two‐step PCR approach is costly as it has to include both the sequence complementary to the sequence overhang, the sequence adapters, and the library indices (Figure 2c). It is worth noting that many labelled index primers will have to be purchased if twin dual‐indices are used to account for incorrect assignment of indices to libraries. This second primer set is, however, applicable across different metabarcoding primer sets and can thereby be used across many metabarcoding studies. For all three approaches, cost‐effectiveness will be increased if the purchased primers are depleted effectively, that is, if they are not only to be used in one small study. The two primer sets in the untagged two‐step PCR approach (Figure 2c) have good potential for being used up, as the first unlabelled metabarcoding primer set can be used across many samples and the second primer set can be used across different metabarcoding primer sets.

### 3.6 Laboratory workload

The one‐step PCR approach is without doubt the quickest method for generating sequence‐ready libraries, as it only requires a single PCR‐step to achieve both amplification and library preparation of the metabarcoding amplicons (Figure 2b). Researchers have used this method in research and commercial scenarios to turn around sequence data in 12–24 h in the field on the iSeq platform (Bunce, unpublished data). In some applications, especially requiring timely interventions, the rapid turnaround time of the one‐step PCR approach may be a consideration. The workload for the two‐step PCR approach and the tagged PCR approach depends, to some extent, on how many sample extracts and PCR replicates are to be processed. If it is a relatively high number, the tagged PCR approach is the quickest due to the library build being performed on pooled amplicons rather than through a PCR step on individual PCR products. However, as with all molecular biological workflows, carefully organised liquid handling and automation provide solutions to high‐throughput studies.

## 4 CHOOSING A METABARCODING APPROACH

It is clear that there is no such thing as a perfect metabarcoding sample‐labelling approach, and that choosing which one is right for a given study or laboratory should be an informed trade‐off of pros and cons balanced to the needs (Table 1). Within metabarcoding studies, those needs can range widely.

Metabarcoding studies range from those that look for one or a few taxa within sample units ( Bohmann et al., 2018) to studies that look for many taxa within sample units (Seersholm et al., 2018), and sample numbers can range from tens (Elbrecht et al., 2017), to hundreds (Galan et al., 2017; Rodgers et al., 2017) or even thousands (Ji et al., 2021; Schnell et al., 2018). The research question and experimental set‐up can require taxonomic identifications to be made within individual samples (Coghlan et al., 2012), while in other studies, taxonomic identifications from pools of individual samples or from a number of samples within, for example, a geographic location is the goal (Grealy et al., 2016; Schnell et al., 2018). Sample types can range from bulk specimen samples consisting of high quality DNA from pools of entire organisms (Tang et al., 2015) to environmental samples in which DNA from target organisms can be fragmented and scarce (Stat et al., 2017). Furthermore, studies differ in how many metabarcoding primer sets are used ‐ from only one ( Bohmann et al., 2011; Drinkwater et al., 2018) to several (De Barba et al., 2014; Drummond et al., 2015; Zhang et al., 2018). Furthermore, the budget for a metabarcoding project will differ between studies, as will whether the metabarcoding primers are to be used in future studies. Lastly, some applications of metabarcoding, such as biosecurity or forensics, will necessitate a “high bar” for data fidelity and controls.

A multitude of combinations of the above metabarcoding study parameters exist, and as demonstrated by this article, the significance of the pros and cons of the metabarcoding approaches will differ with them. For example, while the tagged PCR approach (Figure 2e) may excel in amplifying low abundance templates given the shorter nucleotide additions to the metabarcoding primers than the one‐step PCR primers (Murray et al., 2015; Zizka et al., 2019), the one‐step PCR offers a quicker turnaround (Figure 2b). However, the one‐step PCR strategy comes at the cost of buying long fusion primers, and is only worthwhile if the metabarcoding primers are to be used again.

When choosing a metabarcoding approach, the need for future multiplexing of the metabarcoding primers should be considered. That is, to use several metabarcoding primer sets that target different markers and taxonomic groups within the same PCR reaction to simultaneously screen for many taxonomic groups and thereby keep costs and work load at a minimum (e.g., De Barba et al., 2014). For this, the nucleotide tagged primers in the tagged PCR approach should theoretically be the most applicable, whereas the long additions to the metabarcoding primers in the one‐step and two‐step PCR approaches might be less conducive to multiplexing due to the extensive sequence homology.

Lastly, it should be noted that whatever metabarcoding strategy is chosen, it should be clear from the present article that one should not change workflows within an experiment. Moreover, there is some justified concern within the metabarcoding community that the nuances in metabarcoding workflows makes interlaboratory comparison difficult (Blackman et al., 2019; Murray et al., 2015; Zizka et al., 2019).

## 5 APPLICATIONS ON OTHER SEQUENCING PLATFORMS

Although to a more limited extent, other second generation sequencing technologies than Illumina are used in metabarcoding. For example, Ion Torrent (Thermo Fischer Scientific) and BGI platforms (BGI Genomics) (Braukmann et al., 2019; Forin‐Wiart et al., 2018; Schnell et al., 2018; Yang et al., 2020). These technologies require the addition of sequencing adapters similar to Illumina platforms and have similar labelling structure. Therefore, discussions regarding labelling strategies in the present article are largely applicable to metabarcoding on these other platforms. For example, the one‐step (Schnell et al., 2018) and the two‐step PCR approach (Braukmann et al., 2019; Nota et al., 2019) have been used on the Ion Torrent platform, and the tagged PCR approach has been used on BGI's MGISEQ platform (Yang et al., 2020). Further, third generation technologies yielding long reads have been employed in metabarcoding; Pacific Biosciences (PacBio) (James et al., 2016; Tedersoo et al., 2018) and the portable Oxford Nanopore Technologies MinION sequencer (Karst et al., 2021). These platforms also rely on the addition of sequencing adapters. The high error rate of these platforms (Dohm et al., 2020) compared to Illumina platforms (Stoler & Nekrutenko, 2021) makes correct taxa identification and sample specific labelling difficult. However, solutions to this are being developed (Karst et al., 2021). It is likely that metabarcoding applications will probably follow the platform with the highest sequencing fidelity although in some applications speed and portability may also increasingly become factors in platform choice.

## 6 PERSPECTIVES

All metabarcoding strategies can generate robust data. However, like all laboratory workflows if they are not executed well or are inappropriate for the application, they may lead to flawed data. We advocate that just because PCR is a relatively simple method it does not mean that metabarcoding is simple, and there are many traps in metabarcoding workflows that can trip‐up new users. Here, we have presented an overview of the three main metabarcoding strategies for assessment of biodiversity on Illumina sequencing platforms, and the downstream consequences for the resulting data with regards to cross‐contamination risk, PCR amplification efficiency, chimera formation, tag‐jumping, index‐misassignment, as well as cost and workload. In doing so we wish to enable researchers and practitioners to make an informed choice of which metabarcoding strategy is best suited for their specific study. Ultimately, this is to avoid the worst case scenario: generation of unusable data and wasting a considerable amount of time and money, or even worse making wrong conclusions due to flawed data.

Metabarcoding of environmental DNA has some commonalities with the field of ancient DNA in which low quality and quantity of target DNA is also targeted amongst nontarget, and potentially more abundant, templates. In the early days of ancient DNA studies, PCR‐based techniques, including amplifying already amplified DNA to enhance signals, were used, which caused authentication issues, as amplification of modern templates was mistaken for true ancient signals. This was followed by urgent calls for precautions to ensure reliability and authenticity of ancient DNA sequences (Cooper & Poinar, 2000; Pääbo et al., 2004). Also similarly to the field of ancient DNA, the take‐home message should be that metabarcoding is becoming a self‐critical and self‐correcting field in which technical reliability is promoted and rewarded, with the long‐term benefit of uptake by stakeholders who will employ metabarcoding for environmental management. Reputational setbacks as the result of practitioners not executing their metabarcoding workflows well will probably resonate across a variety of biomonitoring, forensic, and bioseurity applications.

We thus stress the importance of being informed about the pros and cons of the chosen metabarcoding approach with regards to cross‐contamination risk, PCR amplification efficiency, chimera formation, tag‐jumping, index‐misassignment, cost, and workload and to include appropriate quality assurance and quality control measures. This will help ensure that the generated data will facilitate informed data analysis and interpretation. We advocate that metabarcoding publications should include detailed information about the metabarcoding strategy and how its challenges have been taken into account in the laboratory, data processing, and interpretation of results. Furthermore, it may be appropriate to eventually develop a set of metabarcoding guidelines similar to the MIQE guidelines for qPCR (Bustin et al., 2009) to establish standard reporting practises, which would ultimately further increase the power and reliability of metabarcoding.

## Conflict of Interest

The authors declare no conflict of interest.

## Data Availability

Data sharing is not applicable to this article as no new data were created or analyzed in this study.
